# To Compare the Effectiveness of Low-Molecular-Weight Heparin and Unfractionated Heparin in Reducing Lower Limb Girth in Deep Vein Thrombosis

**DOI:** 10.7759/cureus.59449

**Published:** 2024-05-01

**Authors:** Neha Ullalkar, Vedanth M, Sreeramulu PN, D Vaibhavi, Shashirekha CA

**Affiliations:** 1 General Surgery, Sri Devaraj Urs Medical College, Kolar, IND; 2 Surgery, Sri Devaraj Urs Medical College, Kolar, IND

**Keywords:** low-molecular-weight heparin, low-molecular weight heparin (lmwh), bleeding risk, lower limb girth, unfractionated heparin, deep vein thrombosis

## Abstract

Introduction: Treating deep vein thrombosis (DVT) using a once-daily dose of enoxaparin offers greater convenience and the possibility of home-based care for certain patients, as opposed to a continuous infusion of unfractionated heparin (UFH). The study aimed to determine the most cost-effective thromboprophylaxis between low-molecular-weight heparin (LMWH) and UFH for hospitalized patients.

Materials and methods: After obtaining clearance from the institutional ethical committee, the study was conducted in the Department of General Surgery, Sri Devaraj Urs Medical College, over a period of six months. Informed consent was obtained from all 46 patients included in this study. The participants were divided into two groups: group A received LMWH and group B received UFH.

Results: The mean age in group A was 59.8 + 10.6 years and in group B was 54.9 + 12.3 years. There was no significant difference in the girth of the lower limb between the groups during the follow-up period (p > 0.05). In group A, there was a highly significant reduction in lower limb girth from day one to day five (p < 0.0001), day five to day 10 (p < 0.0001), and day one to day 10 (p < 0.0001). In group B, there was no significant reduction from day one to day five (p = 0.06), but there was a significant reduction from day five to day 10 (p = 0.001) and day one to day 10 (p = 0.001).

Conclusion: Treatment with LMWH as an anticoagulant significantly reduced the lower extremity girth and thrombus thickness in cases of DVT when compared to UFH.

## Introduction

Venous thromboembolism (VTE), which usually appears as deep vein thrombosis (DVT) in the lower extremities or as pulmonary embolism, occurs at a yearly rate of one to two cases per 1,000 people [1]. The death rate is significant; approximately 6% of patients with DVT pass away within 30 days, mainly due to pulmonary embolism, and this figure rises to 13% for patients suffering from pulmonary embolism [2]. In patients who receive treatment, approximately 20-50% experience post-thrombotic syndrome (PTS) following DVT, and 3% go on to develop chronic thromboembolic pulmonary hypertension after a pulmonary embolism [3,4].

Heparin varies in molecular size, anticoagulant effectiveness, and pharmacokinetic characteristics. Its molecular weight spans from 3,000 to 30,000 Daltons (Da), averaging around 15,000 Da [5-7]. Administering heparin at a consistent low dose of 5,000 U subcutaneously (SC) every eight or 12 hours serves as an effective and secure preventive measure against VTE in medical and surgical patients at risk. This low-dose heparin regimen decreases the likelihood of venous thrombosis and fatal pulmonary embolism by 60% to 70% [8,9].

For preventing VTE in trauma patients, the recommended medication is low-molecular-weight heparin (LMWH), specifically enoxaparin, administered at a dosage of 30 mg twice a day [10]. Nonetheless, the main prospective randomized clinical trial that showed the effectiveness of LMWH for preventing VTE in trauma patients compared it to a regimen of 5,000 units of unfractionated heparin (UFH) administered every 12 hours. This is in contrast to the more commonly accepted dosing schedule of 5,000 units every eight hours [11]. In a hospital environment, administering LMWH once or twice daily for DVT treatment has been proven to be equally effective and safe as treatment with intravenously infused UFH [12,13].

LMWH is commonly utilized, particularly in cases of cancer-associated thrombosis (CAT). In comparison to UFH, LMWH tends to demonstrate a more consistent dose-response relationship. It possesses a longer half-life, which permits once or twice daily subcutaneous administration. Additionally, LMWH carries a reduced risk of heparin-induced thrombocytopenia and osteoporosis. However, since LMWH is primarily eliminated through the kidneys, its use is not recommended in patients with significant renal impairment. On the other hand, UFH, with its shorter half-life, can be advantageous for patients needing an immediate anticoagulant effect, facilitating rapid reversal when necessary [14].

The global priority for improving patient safety is the prevention of VTE in hospitals, using either low-dose UFH or LMWH. Despite higher costs, LMWHs offer benefits like less frequent dosing and a safer side effect profile [15]. The study aimed to determine the most cost-effective thromboprophylaxis approach for hospitalized patients, including various subgroups. This study compares the effectiveness of LMWH and UFH in the rate of reduction of lower limb girth in DVT, which would further help us in establishing standard guidelines for effective thromboprophylaxis.

## Materials and methods

Study design, sample size, and source of data

The Institutional Ethical Committee, Sri Devaraj Urs Academy of Higher Education and Research approved the study and granted permission to start the study with approval number DMC/KLR/IEC/648/2024-25. This was a retrospective study, which included 46 subjects. We compared hospital-administered adjusted-dose intravenous standard heparin and appropriate home-administered fixed-dose subcutaneous LMWH. We divided the subjects into two groups: group A received LMWH and included 25 study subjects and group B received UFH and included 21 study subjects in the Department of General Surgery, Sri Devaraj Urs Medical College, over a period of six months (January 2023 to June 2023). Measurements were taken over the calf (10 cm below tibial tuberosity) and were done by the primary investigator in all cases. As it was a retrospective study, the sample size taken was according to each individual treating doctor's protocol, hence both groups do not have an equal number of subjects. Table 1 shows the inclusion and exclusion criteria.

**Table 1:** Inclusion and exclusion criteria

Inclusion criteria	Exclusion criteria
Patients who are willing to give written informed consent	Venous thromboembolism in the past 2 years
Age above 18 years	Life expectancy less than 1 year
Patients who are bed-ridden	Patients who have been taking other anti-platelets or anti-coagulants
Prolonged duration of hospitalization	History of allergic reactions to heparin or low-molecular-weight heparin
Fulfilling Wells criteria [16]	Pregnant or lactation women

Method of data collection

After obtaining consent from the patients, the study was conducted. DVT was confirmed through a duplex scan or venography. Patients with confirmed lower limb DVT, eligible for outpatient treatment, were randomly assigned to either receive enoxaparin at a dosage of 1.5 mg/kg once daily subcutaneously for five to 10 days or UFH at an initial intravenous bolus of 5,000 IU, followed by an intravenous infusion of 500 IU/kg/day adjusted to maintain an activated partial thromboplastin time (aPTT) between 1.5 and 2.5 times the normal value for five to 10 days.

Patients underwent a minimum of three follow-up assessments after treatment to measure the circumference of the lower limb at the level of the calf approximately 10 cm below tibial tuberosity on day one, day five, and day 10. Subsequently, they were instructed to return for further examination only if they experienced symptomatic VTE recurrence. Additionally, all patients were contacted, at the very least via telephone, at the conclusion of the follow-up period. Patients in the enoxaparin group were admitted to the hospital based on the physician's judgment, while those in the UFH group were required to be hospitalized for a period ranging from five to 10 days. Every investigator received a randomization plan that outlined the assigned treatment for each patient participating in the study. The investigator recorded the study medication and the corresponding randomization number on a case report form.

Statistical analysis

Data were entered into a Microsoft Excel data sheet (Microsoft Corporation, Redmond, WA) and were analyzed using SPSS version 22 software (IBM Corp., Armonk, NY). Categorical data were represented in the form of frequencies and proportions. Continuous data were represented as mean and standard deviation. The normality of the continuous data was tested by the Shapiro-Wilk test. Student's t-test and repeated measure ANOVA tests were used to compare continuous variables. The chi-square test was used to compare categorical variables. Microsoft Excel and Microsoft Word (Microsoft Corporation, Redmond, WA) were used to obtain bar diagrams. A p-value (probability that the result is true) of <0.05 was considered statistically significant after assuming all the rules of statistical tests.

## Results

The study comprised two groups: group A with 25 subjects and group B with 21 subjects. The mean age in group A was 59.8 ± 10.6 years and in group B was 54.9 ± 12.3 years. There was no significant difference in the age between the two groups (p > 0.05). In group A, the majority were males (16, 64%), and in group B, the majority were females (12, 57.1%). The demographic characteristics are summarized in Table 2.

**Table 2:** Comparison of baseline characteristics Age is expressed as mean ± standard deviation and other variables as frequency and percentage. P < 0.05 was considered significant.

Variables	Group A (n = 25)	Group B (n = 21)	p-value
Mean age (in years)	59.8 ± 10.6	54.9 ± 12.3	0.167
Age groups, n (%)			
35 - 44 years	1 (4%)	5 (23.8%)	0.099
45 - 54 years	4 (16%)	5 (23.8%)	
55 - 64 years	13 (52%)	5 (23.8%)	
65 - 74 years	4 (16%)	5 (23.8%)	
75 - 84 years	2 (8%)	0 (0%)	
>85 years	1 (4%)	1 (4.8%)	
Gender			
Male, n (%)	16 (64%)	9 (42.9%)	0.156
Female, n (%)	9 (36%)	12 (57.1%)	

Student's t-test was applied to compare the girth of the lower limb between the two groups on day one, day five, and day 10 of the follow-up period after therapy. The measurements are summarized in Table 3. There was no significant difference in the girth of the lower limb between the groups during the follow-up period (p > 0.05).

**Table 3:** Comparison of lower limb girth between the two groups The variables are expressed as mean ± standard deviation. P < 0.05 was considered significant.

Lower limb girth (in cm)	Group A (n = 25)	Group B (n = 21)	p-value
On day 1 after therapy	47.5 ± 6.2	45.6 ± 4.6	0.254
On day 5 after therapy	44.9 ± 4.2	47.6 ± 5.4	0.573
On day 10 after therapy	42.6 ± 4.9	42.7 ± 3.8	0.936

**Figure 1:**
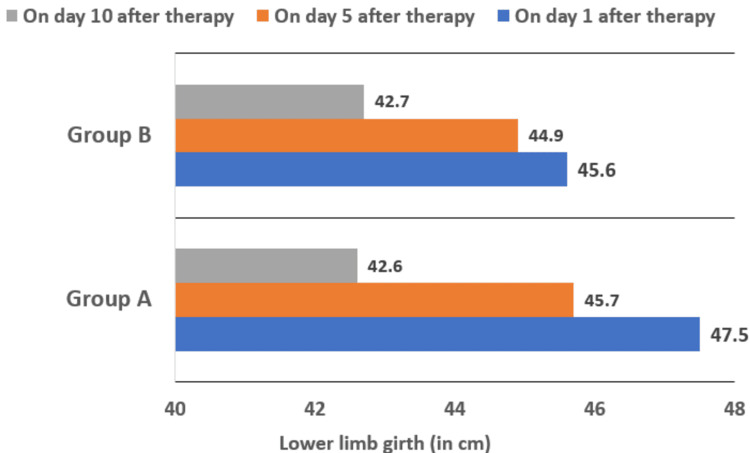
Comparison of mean lower limb girth at various time points between the two groups

To compare the efficacy of LMWH and UFH, we performed repeated measure ANOVA to assess the difference in the lower limb girth over three follow-up time points and compared them in each group. The intragroup analysis is summarized in Tables 4-7. In group A, there was a highly significant reduction in lower limb girth from day one to day five (p < 0.0001), day five to day 10 (p < 0.0001), and day one to day 10 (p < 0.0001). In group B there was no significant reduction from day one to day five (p = 0.06), but there was a significant reduction from day five to day 10 (p = 0.001) and day one to day 10 (p = 0.001). Therefore, the patients who received LMWH had a significant reduction in lower limb girth overall compared to those who received UFH (Figure 1).

**Table 4:** Intragroup analysis of lower limb girth at three time points in group A * P < 0.001 is statistically significant.

Lower limb girth (in cm)	Mean	SD	p-value
On day 1 after therapy	47.5	6.2	<0.0001*
On day 5 after therapy	45.7	5.4	
On day 10 after therapy	42.6	4.9	

**Table 5:** Pairwise comparison of lower limb girth in group A

Lower limb girth (in cm)	Mean difference	95% CI of mean difference
Day 1 – Day 5	1.84	0.858 – 2.822
Day 1 – Day 10	4.96	3.539 – 6.381
Day 5 – Day 10	3.12	2.2 – 4.04

**Table 6:** Intragroup analysis of lower limb girth at three time points in group B * P-value < 0.0001 is significant.

Lower limb girth (in cm)	Mean	SD	p-value
On day 1 after therapy	45.6	4.7	<0.0001*
On day 5 after therapy	44.9	4.2	
On day 10 after therapy	42.7	3.8	

**Table 7:** Pairwise comparison of lower limb girth in group B

Lower limb girth (in cm)	Mean difference	95% CI of mean difference
Day 1 – Day 5	0.76	-0.22 – 1.546
Day 1 – Day 10	2.95	1.276 – 4.629
Day 5 – Day 10	2.19	0.933 – 3.448

## Discussion

In our study, it was shown that the administration of LMWH was effective compared to UFH in treating DVT. Patients in the LMWH group had a slightly extended treatment duration compared to those in the UFH group. Nevertheless, the frequency of hospital visits for each patient was influenced by their individual characteristics rather than their assigned study treatment, which minimizes the potential for bias.

Many clinical research studies indicate that subcutaneous administration of LMWHs at fixed doses can serve as a suitable alternative to standard intravenous UFH for treating DVT [17-21].

The efficacy was demonstrated based on a reduction in lower limb girth after treatment with LMWH and UFH. However, many studies have considered other parameters as indicators of the effectiveness of treatment. Most reviews concerning venous ultrasound following anticoagulation therapy have centered on assessing the risk of recurrent VTE in cases where residual thrombus is present [22-24].

Venous stasis, endothelial damage, and inflammation are commonly observed, creating a heightened tendency for blood clot formation. This prompts the activation of the coagulation process, along with the aggregation of platelets and blood cells, resulting in the development of a thrombus. This thrombus has the potential to block the affected vein partially or completely, leading to conditions like venous stasis, lymphedema, and the potential for surrounding tissue ischemia [25].

LMWHs were initially studied in the mid-1980s to prevent venous thrombosis in high-risk surgical patients. Over time, LMWH has gained extensive recognition for this purpose. In both general surgery and high-risk medical cases, once-daily subcutaneous LMWH is as effective and safe as low-dose subcutaneous UFH administered multiple times a day. LMWH has become the preferred choice for venous thrombosis prevention in major orthopedic surgeries and eligible patients after significant trauma, with a low and comparable risk of bleeding to low-dose heparin [26].

The major limitation of this study is that the sample size is small and did not consider other indicators of resolution of DVT, including thrombus reduction using Doppler ultrasound.

## Conclusions

Our study showed that treatment with LMWH as an anticoagulant has reduced the lower extremity girth and thrombus thickness in cases of DVT when compared to UFH (the initial limb girth of the LMWH team was lesser at the time of initial measurements). This helps in reducing the morbidity and mortality related to lower limb girth and thrombus thickness, which in turn helps in improving the quality of life.
